# The feline sacroiliac joint: An overview of its innervation and morphology

**DOI:** 10.1111/joa.70252

**Published:** 2026-10-02

**Authors:** Alexandra S. Klick, Thomas Grochow, Mirjam Kalusa, Simone A. Fietz, Christoph K. W. Mülling, Nicole Röhrmann

**Affiliations:** ^1^ Faculty of Veterinary Medicine Institute of Veterinary Anatomy, Histology and Embryology, Leipzig University Leipzig Germany

**Keywords:** auricular surface, feline, ilium, innervation, sacroiliac joint, sacrum

## Abstract

The sacroiliac joint (SIJ) serves as a central interface for force transmission from the hindlimbs to the axial skeleton in animals of all species. Its specialized morphology reflects this functional requirement, comprising a smooth auricular surface and a rugose tuberosity covered by interosseous ligaments. In both human and equine medicine, the SIJ is recognized as a primary source of varied back pain. Despite its biomechanical and clinical significance, very limited data exist in small animal medicine concerning the structure, innervation, and clinical relevance of the SIJ. The present study investigated the morphological variation and innervation of the SIJ in the domestic cat (*Felis catus*). Macroscopic assessment of the joint's innervation was performed on 40 specimens, and 20 dorsal sacroiliac ligaments were analyzed microscopically for the presence of nervous tissue. Additionally, the individual components of the joint were scanned three‐dimensionally and measured. The auricular surfaces were subsequently categorized into distinct morphological types. Current investigations have revealed that the dorsal branches of the seventh lumbar and the first sacral spinal nerves course dorsally within the dorsal sacroiliac ligaments, which was confirmed microscopically. Analysis of the size and shape of the articular surfaces showed that the auricular surface can be classified into five different morphological types. No evidence of sexual dimorphism was detected. However, interindividual variation in the size of specific joint components was observed. These findings provide an initial anatomical basis for a better understanding of the SIJ of domestic cats. This enhanced understanding can help optimize diagnostic or therapeutic approaches in small animal medicine. The results therefore may contribute to a preliminary framework for future clinical studies.

## Graphical abstract

The sacroiliac joint of the domestic cat was examined, with a focus on the innervation and morphology of its auricular surface. The study revealed that the last lumbar and the first sacral spinal nerves run within the dorsal sacroiliac ligament, a finding that was confirmed microscopically. Additionally, the auricular surfaces could be classified into five distinct morphological types.

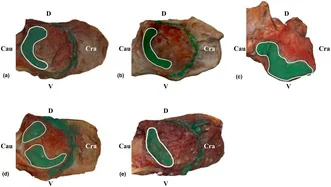

## 1 INTRODUCTION

The sacroiliac joint (SIJ) is a combined synovial and fibrocartilaginous joint connecting the sacrum and the ilium, thereby linking the spine and the pelvis (Evans & de Lahunta, 2013). Functionally, the SIJ acts as a connecting link between the hindlimbs, which generate thrust, and the rest of the body, playing an important role in the animal's quadrupedal gait kinematics. In addition to its role as a connecting link and force transmitter, the SIJ exhibits a shock‐absorbing function. This is particularly relevant for species such as the domestic cat, whose habitual behaviors include jumping, climbing, and running (Dalin & Jeffcott, 1986a; Ellenberger & Baum, 1943; Mijsberg, 1920; Nickel et al., 2004; Salomon et al., 2020). These characteristics necessitate a distinct configuration and organization of the SIJ. In veterinary anatomy, the SIJ is commonly described as comprising an ear‐shaped or crescent‐shaped smooth, ligament‐free auricular surface area, and a rough tuberosity with tight interosseous ligaments (Evans & de Lahunta, 2013; Salomon et al., 2020). Research in equines has provided insight into the configuration of this auricular surface, identifying three principal shape types that may change with age or pathology (Dalin & Jeffcott, 1986a; Jeffcott et al., 1985). Irregularities in the outlines, particularly the presence of extensions of the regular margins of the articular surface, have been observed in older animals and in those with documented SIJ problems. Within the Felidae family, a recent study identified six distinct shape types of the SIJ (Pallandre et al., 2020). The authors attribute the observed diversity of shapes to differences in body mass, hunting behavior, and the resulting biomechanical demands, given that the SIJ plays a central role in power transmission. Additionally, in women, the pelvic girdle and SIJ must accommodate dynamic changes associated with pregnancy and parturition. This sexual dimorphism is evident in the size and shape of the auricular surface of women (Anastasiou & Chamberlain, 2013; Nishi et al., 2020). However, in horses, no sexual dimorphism has been observed (Dalin & Jeffcott, 1986b). Notably, Dalin and Jeffcott (1986b) have demonstrated a positive correlation between the size of the ilium and its corresponding sacrum (Dalin & Jeffcott, 1986b). Beyond morphology, the SIJ is of clinical interest due to its association with pain. In equine medicine, SIJ dysfunction manifests among other problems as lameness, paraspinal muscle tension, and pain upon palpation (Barstow & Dyson, 2015; Dyson & Murray, 2003; Jeffcott et al., 1985; Nagy et al., 2020). In the human clinical setting, SIJ dysfunction is recognized as a cause of lower back pain, which may radiate to the buttocks, thighs, and even the knee along established referred pain patterns (Fortin, Aprill, et al., 1994; Fortin, Dwyer, et al., 1994; Schwarzer et al., 1995; Slipman et al., 2000). In this context, the innervation of the SIJ was also investigated, though there was disagreement in human medicine about the origin of the structures innervating the SIJ (Cox & Fortin, 2004; Grob et al., 1995; Kampsen, 2019; McGrath & Zhang, 2005; Roberts et al., 2014; Steinke et al., 2022; Vilensky et al., 2002). In rats, the innervation of the SIJ has also been demonstrated and these nerves have been shown to transmit nociceptive signals (Murata et al., 2000, 2001, 2007). Similarly, in cats, the presence of nerve tissue has been revealed in the SIJ area, which was classified as potentially nociceptive (Sakamoto et al., 2001).

Despite all these studies mentioned above, there is, to our knowledge, a lack of information regarding the detailed morphological and neuroanatomical features of the SIJ in small animal medicine, particularly in domestic cats. The objective of this study is therefore to document the morphological variation of the feline SIJ and to identify the innervation of the SIJ in cats.

## 2 MATERIALS AND METHODS

To investigate the innervation and anatomy of the sacroiliac joint (SIJ) in cats, 40 (17 spayed female, 3 intact female, and 20 neutered male) adult domestic cats were dissected. The age, possible diseases, and the living conditions of the animals examined were unknown. The animals had an average weight of 4.74 kg and an average crown‐rump‐length of 40.74 cm (see Table S1). Post‐euthanasia, all specimens were immediately frozen (−18°C) and thawed before the anatomical examinations were conducted. All cats were euthanized for reasons unrelated to this study in accordance with animal welfare regulations and applicable legislation, as confirmed by the attending veterinarian. The specimens were donated by veterinary practices in Leipzig to the Institute of Anatomy, Histology, and Embryology for research and teaching purposes. Dissections and three‐dimensional scanning were performed in compliance with prevailing hygiene regulations in the dissecting hall of the Institute of Anatomy, Histology, and Embryology. Animals exhibiting evident trauma to the pelvis, hindlimbs, or spine, as well as those with marked obesity, were excluded from this study. The study was evaluated and approved by the Ethics Committee of the Faculty of Veterinary Medicine, Leipzig University, Leipzig, Germany (EK 7/2021).

### 2.1 Specimen dissection

Initially all cats were weighed, the crown‐rump‐length was measured using a flexible measuring tape, and sex was recorded. Subsequently, the animals were shorn and dissected along the dorsal midline using a scalpel (size No. 4). Magnifying glasses with a magnification of 3.5× were used during dissection to enhance visualization of fine structures.

The skin was reflected laterally to expose the underlying, subcutaneous tissue and fascia. Nerves within the cutaneous area overlying the SIJ were identified and carefully dissected using fine forceps. These nerves were traced ventrally through the middle gluteal muscle and the dorsal intertransversarius muscle of the tail, towards the spine. The spinal canal and the spinal cord of 19 cats were exposed to determine the exact origin of the nerves found and traced back from the skin. In specimens in which the vertebral canal was not opened, the nerves were traced to the intervertebral and dorsal sacral foramen. The documentation exclusively pertained to the presence of nerves that were observed to be passing through the dorsal sacroiliac ligament. To achieve precise localization of the origins of the dorsal cutaneous branches, the spine was divided into segments. The basis of this division was the length of the spinous processes and the spaces between them (Figure 1). The nomenclature of the regions of the spinous processes was determined by their anatomical proximity to the corresponding vertebrae. Interspinal areas are named after the two adjacent vertebrae in their immediate vicinity. In the process of identifying the origin of a nerve, a perpendicular line was drawn medially from its skin attachment to the corresponding spinous process or the area between those, and the corresponding segment was then documented.

**FIGURE 1:**
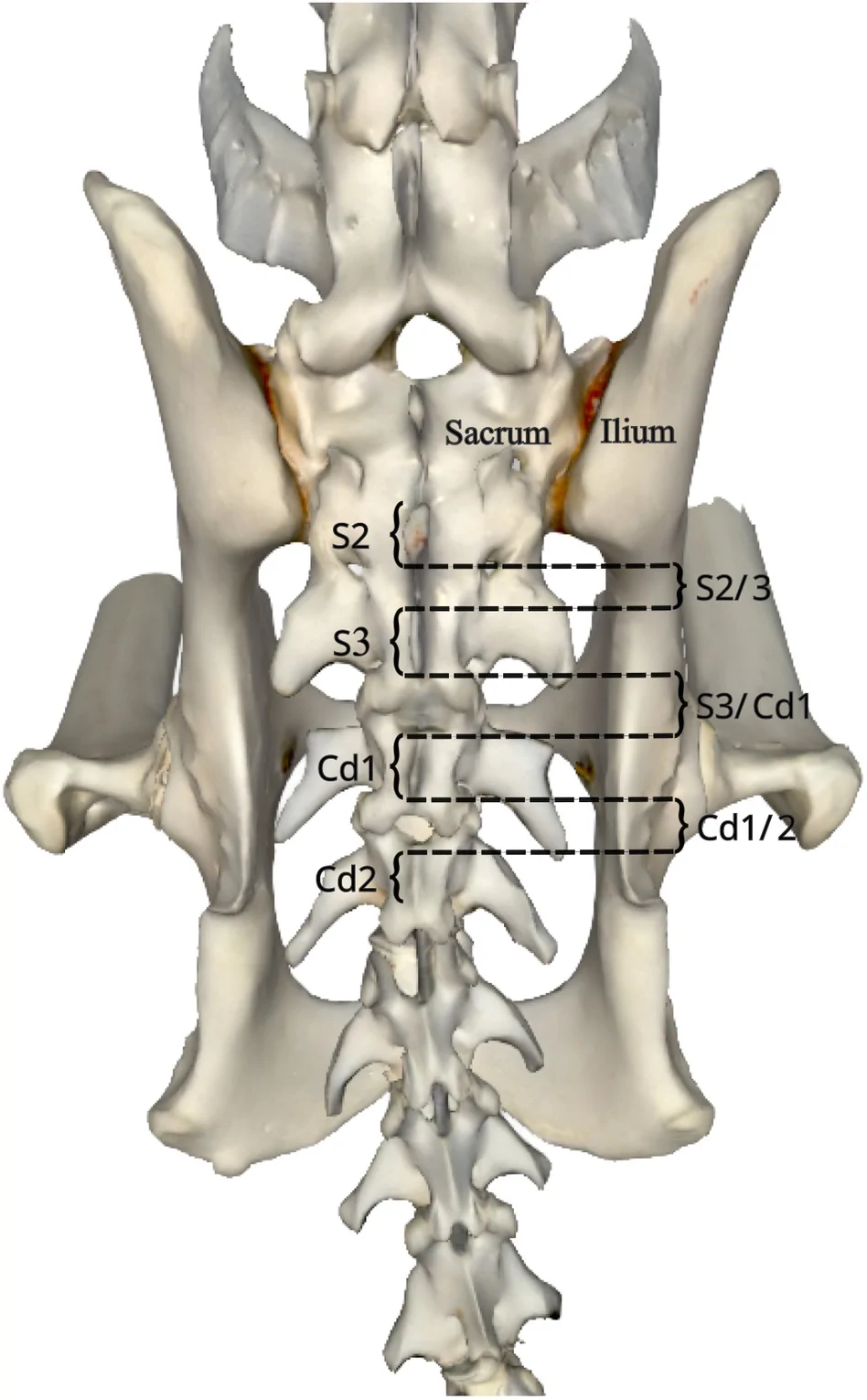
Schematic illustration of the division according to the length of the spinous processes in the regions where the nerves attach to the skin. S2: Region of the second sacral spinous process; S3: Region of the third sacral spinous process; Cd1: Region of the first caudal spinous process; Cd2: Region of the second caudal spinous process; S1/S2: Area between the first and second sacral spinous processes; S2/S3: Area between the second and third sacral spinous processes; S3/Cd1: Area between the third sacral and first caudal spinous processes; Cd1/Cd2: Area between the first and second caudal spinous processes.

### 2.2 Histological analysis

After completion of the dissection of the dorsal branches of the spinal nerves, the right and left dorsal sacroiliac ligaments located between the cranial margins of the SIJ and the first sacral spinous process were excised from ten randomly chosen cats and processed for histological examination. The 20 samples were fixed in 4% buffered formalin for 48–72 h at room temperature. Subsequently, tissues were dehydrated and embedded in paraffin. From each tissue block, four serial sections (1 μm) were cut and deparaffinized in xylene followed by isopropanol, a descending ethanol series (96% to 70%) and distilled water. Two of these four cuts were stained with hematoxylin and eosin (H&E) (Carl Roth GmbH + Co. KG; Karlsruhe, Germany). The remaining two sections were stained using picrosirius red according to Constantin (Winter et al., 2004). H&E is a standard histological staining technique to evaluate the overall tissue morphology and to establish structural orientation across the sections. To facilitate more precise differentiation between the various tissue types, and to enable more accurate differentiation of bundles of nerve fibers from the surrounding dense connective tissue picrosirius red according to Constantin was utilized. Histological identification of nerves was conducted using established morphological criteria. In H&E sections, these structures were defined by bundles of faintly stained, parallel nerve fibers with characteristic flattened Schwann cell nuclei situated between them and surrounded by epineuria. In picrosirius red according to Constantin staining, smooth muscle tissue is observed to appear yellow, while collagen fibers are seen to appear red and elastic fibers appear bright violet. Conversely, the nuclei manifest as brown. For this procedure deparaffinized sections were then treated with 3% acetic acid and stained for 30 min in 1% Alcian blue solution (Dr. K. Hollborn & Söhne GmbH & Co. KG, Leipzig, Germany). After rinsing in tap water for 3 min, the samples were briefly immersed in 70% ethanol and subsequently stained in aldehyde fuchsin solution for 15 min (Dr. K. Hollborn & Söhne GmbH & Co. KG, Leipzig, Germany) followed by another immersion in 70% ethanol. The sections were rinsed in tap water for 3 min and stained with Weigert's hematoxylin for 5 min. After washing in distilled water followed tap water for 5 min, the sections were stained for 30 min in 0.1% picrosirius red solution according to van Gieson (Dr. K. Hollborn & Söhne GmbH & Co. KG, Leipzig, Germany). Following staining, all sections were dehydrated through an ascending alcohol series, cleared in Xylene, and mounted using ROTI® Histokitt (Carl Roth GmbH + Co. KG; Karlsruhe, Germany). As described above, four serial sections were prepared per level, and this procedure was repeated four to eight times for each ligament specimen, resulting in 16–32 sections per specimen. Histological slides were examined and photographed using an Axio lab 5 microscope equipped with an Axiocam 208 color (Carl Zeiss Microscopy Deutschland GmbH, Oberkochen, Germany). Overview images were digitized using an Axio Scan 7 (Carl Zeiss Microscopy Deutschland GmbH, Oberkochen, Germany). Subsequent image processing of the images was performed using Zen 3.13 software (Carl Zeiss Microscopy Deutschland GmbH, Oberkochen, Germany). Images were adjusted using Adobe Photoshop 2025 (version 26.0, Adobe Inc., San José, CA, USA) for visualization purposes only. The graphical templates and vector‐based labeling were developed using Adobe Illustrator 2025 (version 26.0, Adobe Inc., San José, USA).

### 2.3 3D‐scan, joint measurements and form

After completion of nerve dissection and ligament removal, the SIJs were carefully opened using a scalpel. The individual components of the joint were scanned in three dimensions using an Artec Spider 3D‐scanner (Artec 3D, Senningerberg; Luxemburg, accuracy 0.05 mm). Two areas were defined for the measurement of the joint: the total joint surface and the auricular surface. The total surface area comprises the auricular surface and the morphologically different tuberosity. The auricular surface is smooth, ligament‐free, and slightly elevated, whereas the tuberosity is covered with ligaments. To improve contrast and enhance edge detection for the 3D‐scanner, the edges of the joint and auricular surface of the joint were marked with green acrylic paint. The acquired scans were processed using Artec Studio software versions 18 and 19 (Artec 3D, Senningerberg; Luxemburg). For each dataset, the background was removed prior to alignment of the scans. To generate a 3D model, it was necessary to register all scans, remove any outliers, and subsequently fuse the scans. For morphometric analysis, the total joint surface was delineated along the marked margins using the eraser tool of the software. The resulting surface area could then be quantified using the software's measuring tool. To determine the size of the auricular surface, this area was separately segmented and measured using the measuring tool. The outlines of the scanned and segmented auricular surfaces were subsequently classified into different shape types by one observer. The right and left sides, as well as the sacrum and ilium, were analyzed independently for all parts of the joint. The classification was based on the subdivision into dorsal and ventral limbs introduced by Jesse et al. (2017) and Pallandre et al. (2020) (Figure 2).

**FIGURE 2:**
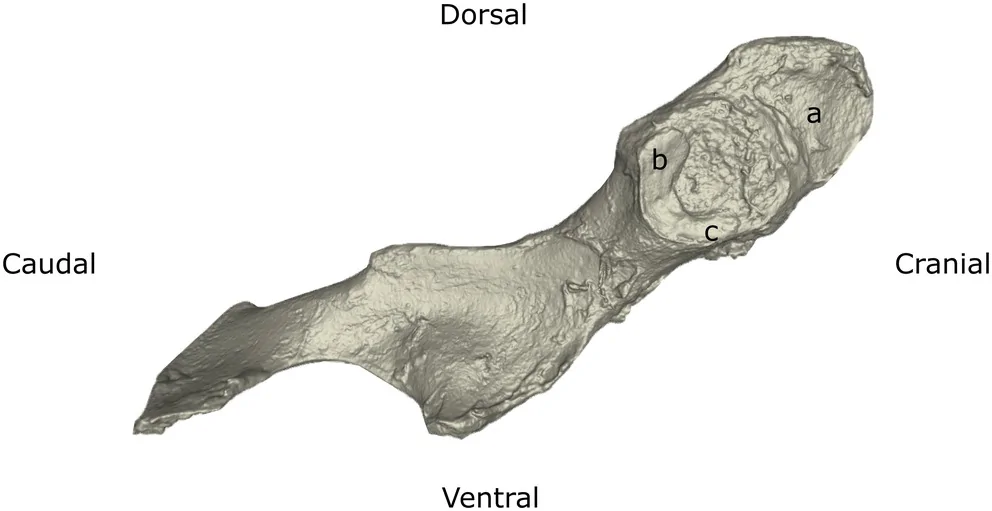
3D scan of the medial view of the left hip bone of a domestic cat presenting the subdivision of the auricular surface into dorsal and ventral limb. a: Iliac wing; b: dorsal limb of the auricular surface; c: ventral limb of the auricular surface.

### 2.4 Statistical analysis

Statistical analyses were performed using the software Origin Pro 2019 (OriginLab, Massachusetts, USA). The normality of the auricular surface size and total joint size for both sacrum and ilium of left and right side as well as of the crown‐rump‐length was assessed by the Shapiro–Wilk test. Joint surface area measurements were normally distributed. The data collected on crown‐rump‐length could not be identified as normally distributed. Intergroup comparisons of the auricular surface size and total joint size were conducted using a paired‐samples *t*‐test. Comparison of the individual joint parts of male and female was done with an independent two‐sample *t*‐test. The level of significance was set at *p* < 0.05 (*α* = 0.05). To assess the correlation between the crown‐rump‐length and surface area Spearman correlations was used. To assess the correlation between the surface area of the sacrum and the corresponding ilium Pearson correlations was used.

To quantify bilateral symmetry between the left and right SIJ surfaces, the Symmetry Index (SI) according to Robinson et al. (1987) was calculated using the following formula:

$$\mathrm{SI}=\frac{\left(\left|X_{\text{left}}-X_{\text{right}}\right|\right)}{\left(0.5*\left(X_{\text{left}}+X_{\text{right}}\right)\right)}\times 100$$

An SI value of 0% represents perfect symmetry, whereas increasing values reflect greater asymmetry between the sides.

Python was used for the graphical visualization of the nerve pathway using sanky diagrams (Python Software Foundation, Delaware, USA).

## 3 RESULTS

### 3.1 Innervation of the dorsal sacroiliac ligaments

#### 3.1.1 Macroscopic examination

Nerve branches coursing within the ligamentous apparatus of the SIJ were detected in all examined cats. The focus was on the dorsal branches of the seventh lumbar (L7) and first sacral (S1) spinal nerves. After emerging from the intervertebral foramen, the dorsal branch of L7 runs dorsally along the cranial edge of the SIJ within loose connective tissue. Upon reaching the dorsal sacroiliac ligament, the nerve branch continued either superficially or deep within the ligament in a lateral direction. In this latter location, the nerve branch reached the middle gluteal muscle, and from there, it extends as the cranial clunial nerves, with variable caudal distribution (Figures 3 and 4). In cat number one, the right dorsal branch of L7 was torn and could therefore not be traced further.

**FIGURE 3:**
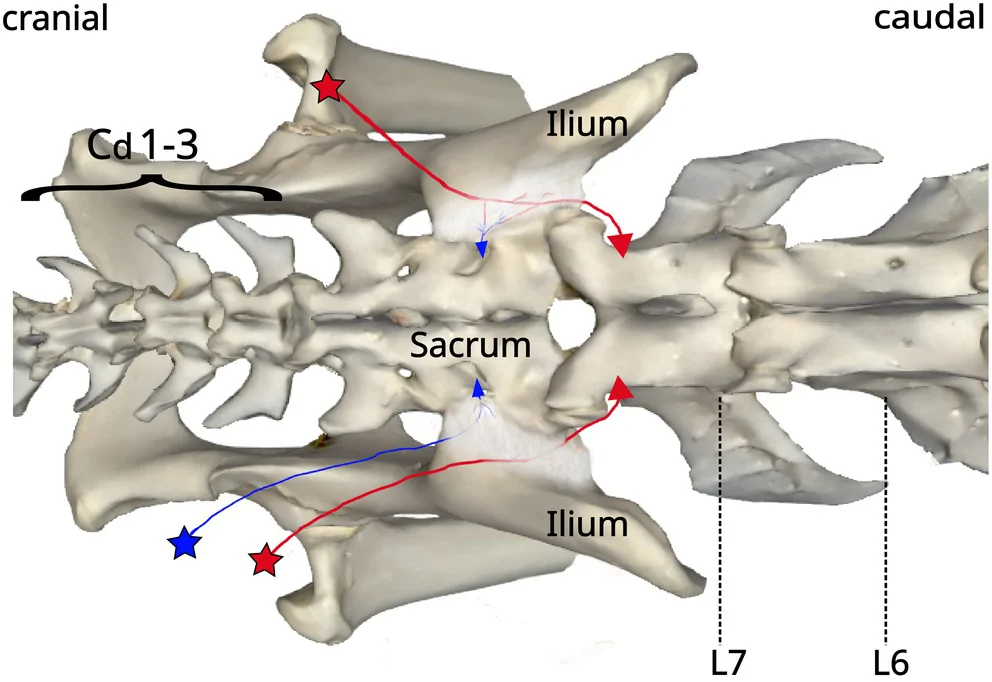
Schematic illustration of the anatomical course of the seventh lumbar and first sacral dorsal nerve branches within the sacral region. L6: sixth lumbar vertebra; L7: seventh lumbar vertebra; Cd 1–3: First to third caudal vertebrae; Red arrow: Dorsal nerve branch of L7; Blue arrow: Dorsal nerve branch of S1; Star: Nerve attachment in the skin.

**FIGURE 4:**
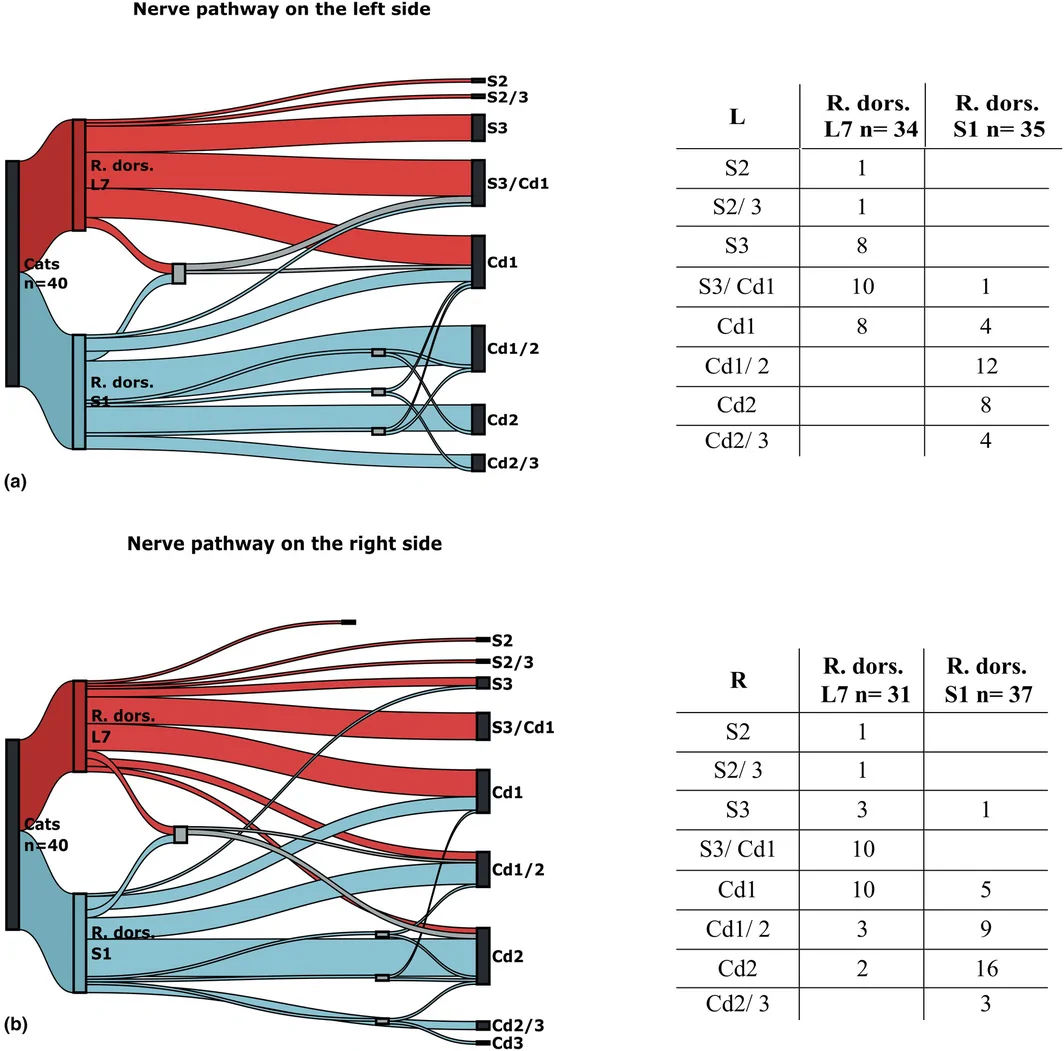
Sankey plots with corresponding tables illustrating nerve pathways on the left (a) and right (b) sides. The Sankey plot illustrates the distribution flow from left to right: The initial single node on the left represents the total number of identified nerves of the examined cats (*n* = 40). This node splits into two color‐coded streams indicating the dorsal branch (red for seventh lumbar and blue for first sacral dorsal branch). From these regional nodes, multiple smaller streams branching to the right illustrate the cutaneous termination points of the nerves at the level of the respective spinous processes or areas between. The width of each stream is strictly proportional to the number of nerve branches that enter each zone. The grey streams represent the converging branches. R. dors. L7: Dorsal branch of L7; R. dors. S1: Dorsal branch of S1. S2: Region of the second sacral spinous process; S3: Region of the third sacral spinous process; Cd1: Region of the first caudal spinous process; Cd2: Region of the second caudal spinous process; S1/S2: Area between the first and the second sacral spinous processes; S2/S3: Area between the second and third sacral spinous processes; S3/Cd1: Area between the third sacral and first caudal spinous processes; Cd1/Cd2: Area between the first and second caudal spinous processes, Cd2/Cd3: Area between the second and third caudal spinous processes.

The dorsal branch of S1 emerged from the first dorsal sacral foramen and proceeded within the loose and tight connective tissue of the dorsal ligamentous apparatus of the SIJ. Fine nerve branches extended into the dorsal sacroiliac ligaments. This branch then coursed superficially over the dorsal sacroiliac ligaments and extended caudolaterally through the dorsal intertransversarius muscle of the tail, continuing to the skin as the middle clunial nerve (Figure 3). Anastomotic connections between the dorsal branches of L7 and S1 were consistently observed.

Variations in the pattern of SIJ innervation were noted in several cats. On the right side, a total of five cats exhibited innervation exclusively from the dorsal branch of S1, whereas two cats exhibited innervation exclusively from the dorsal branch of L7. In cat number 20, no nerve branches could be identified in the SIJ region on the right side. On the left side, four cats demonstrated innervation from the dorsal branch of S1 exclusively, while three animals exhibited nerve branches originating from L7. In two animals, no nerve branches were found on the left side. Some of the dorsal cutaneous branches of S1 split into two in the skin area. Moreover, in three cases, the dorsal branch of L7 and S1 converged to form a common trunk prior to their cutaneous distribution bilaterally (Figure 4 and Table 1).

**TABLE 1:** Variations of nerve branch origins of the feline SIJ on the right and left sides.

Side	R. dors. L7	Rr. dors. L7 & S1	R. dors. S1	None
Right	2	32	5	1
Left	3	31	4	2

*Note*: L7: R. dors. L7: Dorsal branch of L7; Rr. dors. L7 & S1: Dorsal branches of L1 and S1; R. dors. S1: Dorsal branch of S1.

#### 3.1.2 Microscopic examination

Histological examination of the ligamentous tissue revealed dense connective tissue interspersed with strands of loose, vascularized connective tissue, as well as areas of muscle and adipose tissue (Figure 5). Nerves were detected in 16 of the 20 samples examined. In three left side samples and one right side sample, nervous structures could not be clearly verified histologically. The nerves were observed in both transverse and longitudinal sections. However, all nerve fibers examined showed myelination.

**FIGURE 5:**
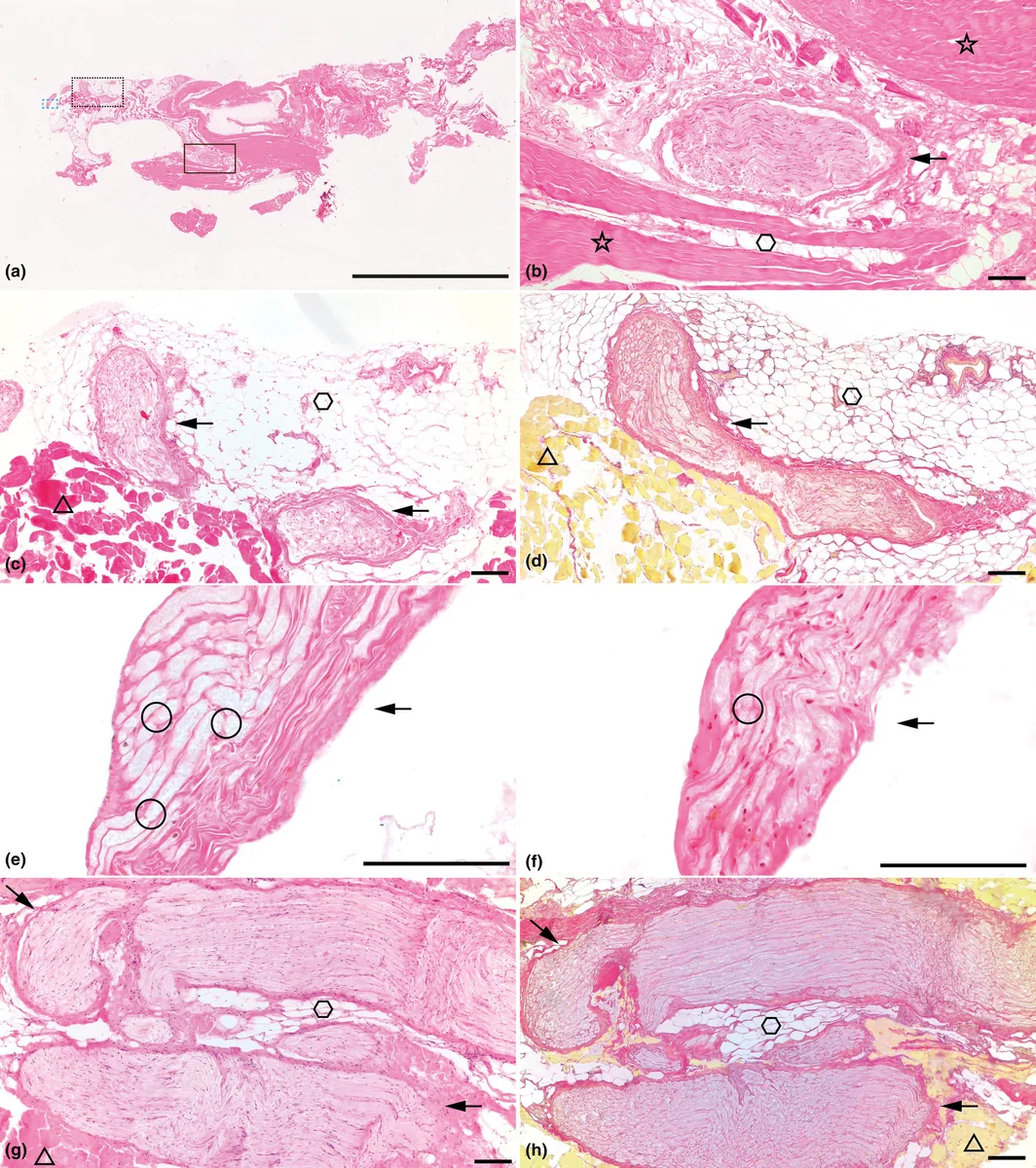
Histological sections of the dorsal sacroiliac ligamentous tissue containing dense, muscle and adipose tissue as well as nerve fibers. (a) Overview showing general distribution of connective tissue and embedded bundle of nerves (H&E). (b) Higher‐magnification of a nerve fibers within the black bordered box in (a) (H&E; 10×). (c, d) small bundles of nerve fibers within the dotted square in (a) (c: H&E, 10×; d: Picrosirius red, 10×); (e, f) Ranvier nodes within nerve corresponding to the box area outlined by the blue dotted square in (a) (e: Picrosirius red, 40×; f: H &E, 40×); g, h: Large nerve exhibiting both longitudinal and transverse axonal orientations (g: H&E, h: Picrosirius red; 10×). Scale bar a: 500 μm, Scale bar b–h: 100 μm; Arrow: nervous tissue; triangle: muscle tissue; Hexagon: adipose tissue; star: dense connective tissue; Circle: Ranvier nodes.

### 3.2 Statistical analysis of joint size

Across all specimens, the mean auricular surface of the ilium, both with respect to the auricular surface area and the total joint surface, exceeded that of the sacrum on both sides. Spearman correlation analyses demonstrated a weak positive correlation (*r* ≤ 0.33) between crown‐rump‐length and surface area (Table 2). By contrast, a strong positive correlation was observed between the surface area of the sacrum and the corresponding ilium (*r* ≥ 0.878) (Figure 6a,b and Table 3). Body weight demonstrated no correlation with either total joint surface area or auricular surface area. Comparison of the right and left sides across all specimens revealed no statistically significant differences in mean auricular surface or total joint surface area. However, side‐to‐side differences were observed at the individual level (Figure 7). The SI showed a considerable variation around 0, with mean values ranging from 5.56 to 10.78 (Figure 7 and Table 4). No statistically significant disparities were identified in mean joint dimensions between male (*n* = 20) and female (*n* = 20) cats (Table S2).

**TABLE 2:** Spearman correlation coefficients (*r*) and corresponding *p*‐values for non‐normally distributed parameters and variations.

Variable 1	Variable 2	Correlation (*r*)	*p*‐value
CRL	I AS	0.27	0.016
CRL	S AS	0.33	0.003
CRL	I TJ	0.23	0.036
CRL	S TJ	0.2	0.077

Abbreviations: AS, auricular surface; CRL, crown‐rump‐length; I, ilium; S, sacrum; TJ, total joint surface.

**FIGURE 6:**
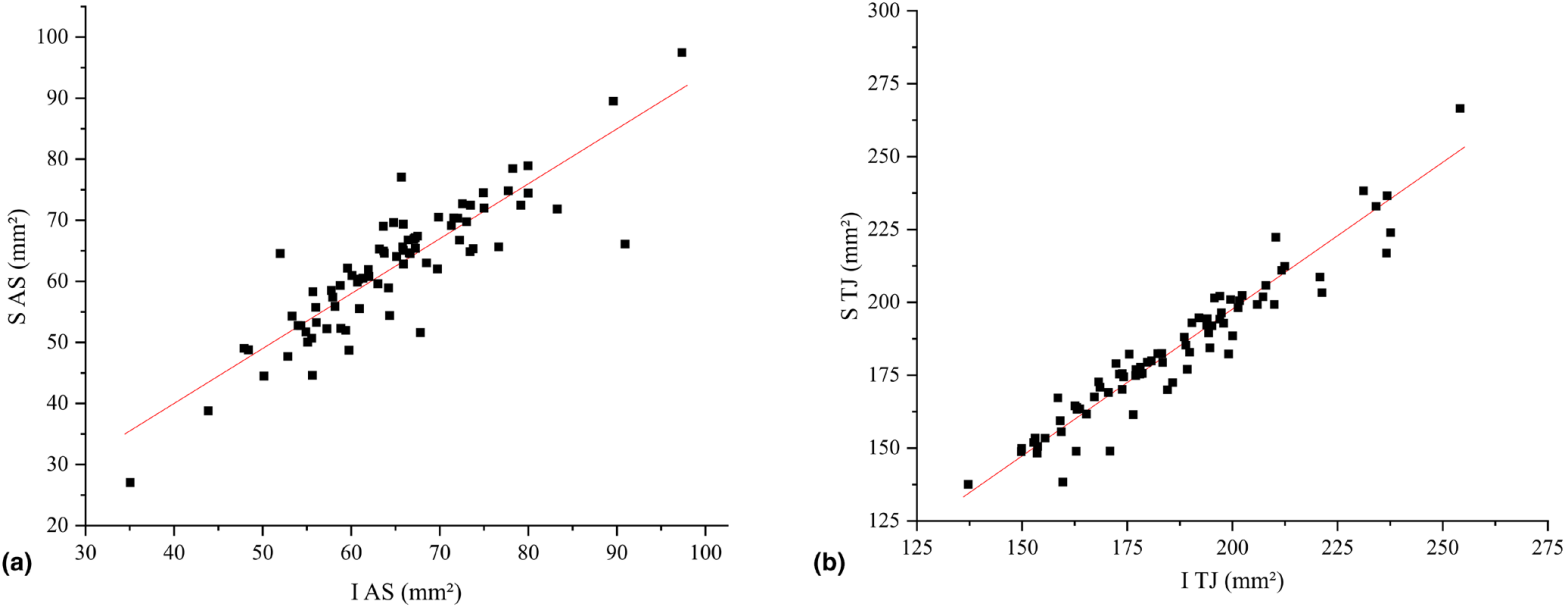
Scatter plots illustrating the correlation between the auricular surface areas of the ilium and sacrum. (a) Correlation between the total joint surface areas of ilium and sacrum. (b) Correlation between the auricular surface areas of ilium and sacrum. AS, auricular surface; I, ilium; S, sacrum; TJ, total joint surface.

**TABLE 3:** Pearson correlation coefficients (*r*) and corresponding *p*‐values for non‐normally distributed parameters and variations.

Variable 1	Variable 2	Correlation (*r*)	*p*‐value
I AS	S AS	0.88	<0.001
I TJ	S TJ	0.96	<0.001

Abbreviations: AS, auricular surface; CRL, crown‐rump‐length; I, ilium; S, sacrum; TJ, total joint surface.

**FIGURE 7:**
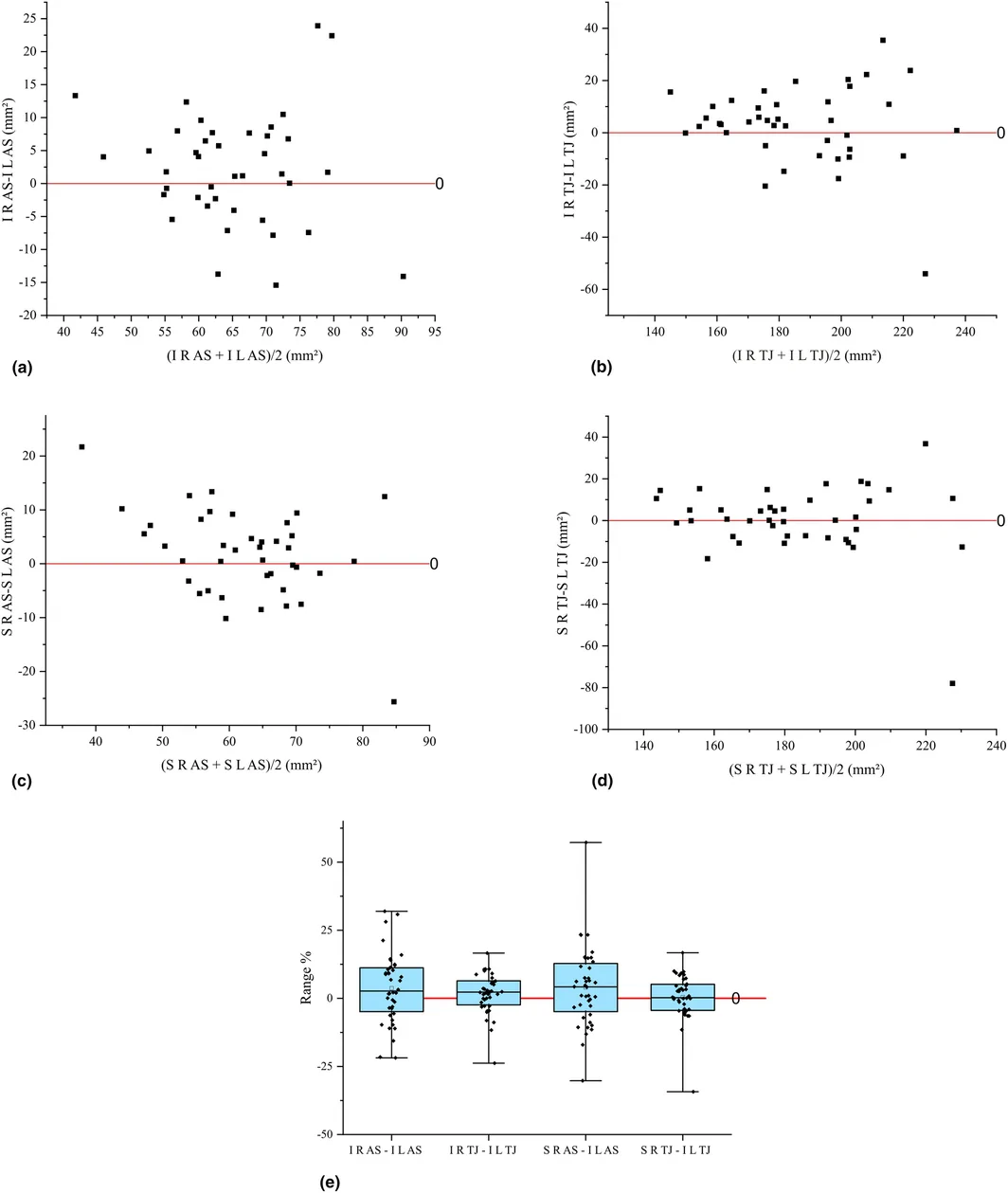
Bland–Altman plots and boxplot illustrating bilateral differences in auricular and total joint surface areas. Bland–Altman plots demonstrate agreement between the surface areas of the right (R) and left (L) sacroiliac joints. The *y*‐axis represents the absolute difference between sides (R − L), while the *x*‐axis shows the mean surface area of both sides combined ((R + L)/2). (a) Difference in auricular surface area of the ilium. (b) Difference in total joint surface area of the ilium. (c) Difference in auricular surface area of the sacrum. (d) Difference in total joint surface area of the sacrum. (e) Boxplot illustrating the Symmetry Index (SI) in percent across the right and left joint surfaces. AS, auricular surface; I, ilium; S, sacrum; SI, Symmetry Index; TJ = total joint surface. Red line: Zero line, Whiskers: Min/Max.

**TABLE 4:** Descriptive statistics of the Symmetry Index (SI) for the sacroiliac joint surface area.

SI	Mean	SD	Median	Minimum	Maximum
	%	%	%	%	%
I R AS—I L AS	10.37	8.05	9.32	0.07	31.98
I R TJ—I L TJ	5.70	4.89	4.60	0.01	23.80
S R AS—S L AS	10.78	10.41	8.17	0.37	57.21
S R TJ—S L TJ	5.56	5.94	4.59	0.05	34.28

*Note*: Data are presented as mean, standard deviation (SD), median, and range (minimum and maximum values). *n* = 40.

Abbreviations: AS, auricular surface; I, ilium; L, left; R, right; S, sacrum; TJ, total joint surface.

### 3.3 Joint shape

The auricular surfaces of the sacrum and ilium involved in the formation of the SIJ, on both the right and left sides, were divided into a dorsal and a ventral limb (Figure 2). Consequently, five different shape types could be identified in domestic cats (Figure 8). Type 1 (“auricular‐shaped”) was characterized by a distinctly wider and more prominent dorsal limb compared with the ventral limb. This type was found in 58.13% (93 cases) of the examined animals. Type 2 (“crescent‐shaped”) showed a ventral and dorsal limb with approximately the same width and resembled a C‐shape. This Type was identified in 28.13% (45 cases) of cats. Type 3 (“spatula‐shaped”) was characterized by no limbs, an irregular outline and was found on the right sacrum of one cat (0.63%). Type 4 (“bifoliate‐shaped”) was characterized by a complete division of the surface into two parts. This Type was identified in 8.13% (13 cases) of the joint surfaces examined. Type 5 (“straight‐shaped”) showed no curvature or indentation and therefore no limbs. 5% (8 cases) of the joint surfaces examined could be assigned to this type. In most specimens, the morphology of the iliac auricular surface corresponded closely to that of the sacral surface with no difference between the right and left sides.

**FIGURE 8:**
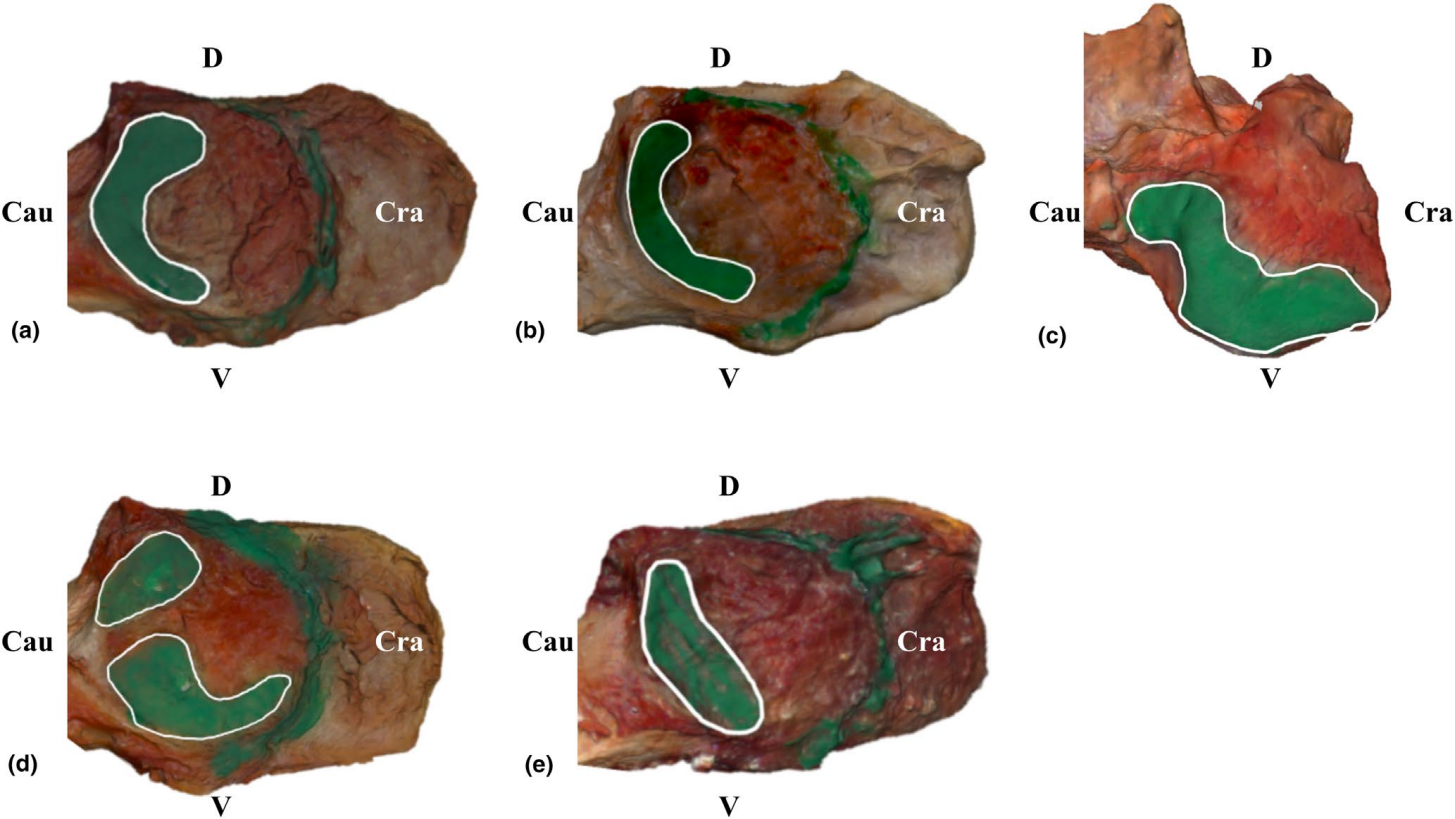
Representative examples of the different auricular surface outline shapes. The auricular surfaces are highlighted with green paint and framed by a white contour line. Pictures (a), (b), (d), and (e) show the auricular surface of the left ilium. Picture (c) shows the auricular surface of the right side of the sacrum. (a) Type 1 (“auricular‐shaped”); (b) Type 2 (“crescent‐shaped”); (c) Type 3 (“spatula‐shaped”); (d) Type 4 (“bifoliate‐shaped”); (e) Type 5 (“straight‐shaped”). Cau, caudal; Cr, cranial; D, dorsal; V, ventral.

## 4 DISCUSSION

The SIJ is a key focus of many current and previous studies in human medicine. The SIJ has been extensively studied in humans, particularly in relation to lower back pain and SIJ referred pain patterns. However, in veterinary medicine, there are still gaps in knowledge regarding the SIJ's anatomy, innervation, and pain‐inducing potential. Therefore, the aim of this study was to examine the macroscopic anatomy and innervation of the SIJ in domestic cats in more detail.

### 4.1 Innervation of the dorsal sacroiliac ligaments

The macroscopic and microscopic findings indicate that the dorsal sacroiliac ligaments are primarily innervated by the dorsal branches of the seventh lumbar and first sacral spinal nerves. The anastomoses found between dorsal branches of the lumbar and sacral spinal nerves are regarded as common anatomical findings, as described in both veterinary and human literature (Cox & Fortin, 2004; Evans & de Lahunta, 2013; McGrath & Zhang, 2005). Comparison with studies in human medicine and other animal species reveals both similarities and differences. Some studies describe dorsal innervation of the human SIJ originating exclusively from varying numbers of sacral segments (Grob et al., 1995; McGrath & Zhang, 2005), whereas others report additional innervation mostly from the dorsal branch of the lumbar spinal nerves (Cox & Fortin, 2004; Kampsen, 2019; Roberts et al., 2014). By contrast, Steinke et al. (2022) have demonstrated that nerves do not reach the SIJ but rather that branches of L5 to S1 pass through the inner sacroiliac ligaments and that branches of S1 to S4 pass through the outer sacroiliac ligaments.

In rats, the SIJ innervation has been reported to arise from L4 or L5 to S2, demonstrating similarities to human anatomy (Murata et al., 2000, 2001). Sakamoto et al. (2001) have previously utilized electrophysiological techniques in cats to demonstrate that the SIJ and the surrounding ligaments and muscles contain mechanosensitive afferent units originating from the fourth to sixth lumbar spinal nerves. The underlying factors contributing to the observed variations in the number of innervating nerves and their origin are multifaceted and not fully elucidated. One possible explanation for the discrepancies is the anatomical variation between species, particularly regarding the number of lumbar and sacral vertebrae. Another potential explanation may lie in methodological differences, particularly in the dissection areas and the examination methods employed. For instance, in the study by Sakamoto et al. (2001), only the dorsal rootlets of L4–L6 were examined. In the present study, the skin in the dorsocaudal area of the SIJ was incised and then dissected ventrally. This approach enabled the targeted investigation of cutaneous branches in a specific area above the SIJ. Expanding the investigated areas in either study could potentially reveal additional nerve branches in the lumbar and sacral regions. Furthermore, the evaluation of nerve tissue in the current study was performed by macroscopic and microscopic observations, whereas Sakamoto et al. (2001) used an electrophysiological approach. Although both investigations are generally suited for the verification of the innervation of the SIJ, their methodological differences may result in discrepancies, particularly with regards to functional fiber types and regional distribution patterns. Despite these differences, the fundamental principle of SIJ innervation via dorsal branches of the lumbar and sacral spinal nerves remains consistent across species.

Myelinated nerve fibers are characterized by axons, which are surrounded by a myelin sheath, formed by Schwann cells. Among the individual Schwann cells, there are small gaps in the sheath, called Ranvier nodes (Liebich, 2010; Welsch, 2006). Myelinated nerve fibers are known to transmit a variety of stimuli to the spinal cord, including mechanical, thermal, and nociceptive signals (Breves et al., 2022). The histological examination of the ligament samples in this study demonstrated the presence of Ranvier nodes and, consequently, the presence of small bundles of myelinated nerve fibers in 16 of 20 samples (Figure 5). The missing evidence of SIJ innervation in the four samples can be attributed to methodological factors. The histological analysis was confined to a specific area of the tissue. Hence, it is possible that nerve fibers were present in unsectioned parts of the samples. Additionally, the dimensions of the nerve fibers might have been below the resolution limit of standard light microscopy. In such cases, their detection would have necessitated the implementation of specialized immunohistochemical staining methods. Sakamoto et al. (2001) classified the nerves in the SIJ and adjacent tissues as high threshold Group III, suggesting a nociceptive function. This might suggest that some of the nervous structures delineated in this study constitute nociceptive afferents. However, a definitive validation of nociceptive nerve fibers is lacking here, but could be achieved, for instance by using more specific functional or immunohistochemical markers in future studies.

Just as numerous studies in human medicine have demonstrated that the SIJ can be a source of lower back pain or referred pain, SIJ dysfunction in equine medicine manifests through diverse clinical signs (Barstow & Dyson, 2015; Dyson & Murray, 2003; Fortin, Aprill, et al., 1994; Fortin, Dwyer, et al., 1994; Jeffcott et al., 1985; Nagy et al., 2020; Schwarzer et al., 1995; Slipman et al., 2000). These variable clinical manifestations of pain caused by the SIJ can be explained by the concept of referred pain, which typically occurs within regions sharing the same segmental innervation (Bogduk, 2009). Given the segmental diversity of SIJ innervation, referred pain zones may be extensive and heterogeneous (Grob et al., 1995; Slipman et al., 2000). The anatomical identification of nerve branches in the dorsal SIJ region provides a structural basis for future microscopical, functional, and clinical studies assessing whether the feline SIJ can contribute to pain, altered locomotion, or clinical signs.

### 4.2 Joint shape

In addition to the innervation of the SIJ, human medicine pays attention to the macroscopic shape of the auricular surface, for which three main shapes have been described (Jesse et al., 2017). In veterinary textbooks, the auricular surface is generally described across species as either auricular‐shaped (Dyce et al., 2010; Ellenberger & Baum, 1943; Salomon et al., 2020) or crescent‐shaped (Evans & de Lahunta, 2013). Comparably, research on healthy and clinically normal horses also showed three main types (Dalin & Jeffcott, 1986a). In contrast to the studies on humans and horses, and the views expressed in veterinary textbooks, this study was able to demonstrate not only similar diversity of forms but also morphological characteristics in domestic cats, as described by Pallandre et al. (2020). The overall similarity in auricular surface morphology can certainly be attributed to the anatomical similarities among feline species. The differences observed between studies as well as the considerable large variation in shape may have multiple explanations. Pallandre et al. (2020) attributed the shape of the auricular surface to differences in predatory behavior and the resulting forces and movements involved. However, the diversity in auricular surface shape in domestic cats might not be explained by predatory behavior alone but could potentially be influenced by different locomotion patterns. Domestic cats are kept as both outdoor and indoor pets, with varying degrees of freedom of movement. Differences in locomotion behavior, including hunting, jumping, and climbing, could influence the mechanical loading and morphological development of the auricular surface shape of the SIJ. Additionally, the diversity of shape, particularly irregular outlines, or curvature might potentially be linked to age‐related or pathological changes. For example, Dalin and Jeffcott (1986b) reported age‐related alterations to the shapes of the auricular surface of the SIJ in horses. Elderly horses exhibited irregular outlines and atypical joint shapes more frequently. Furthermore, horses with poor performance and low‐grade hindlimb lameness have been shown to display abnormal joint outlines compared to clinically healthy individuals (Jeffcott et al., 1985). Similarly, Jesse et al. (2017) proposed a possible association between auricular surface shape and lower back pain or SIJ pathology. However, since living conditions, age, and medical background of the animals examined in the current study were unknown, any assessments regarding a potential impact of age‐ or disease on SIJ morphology in domestic cats should be regarded with reservation.

### 4.3 Joint size

In this study, a weak correlation between crown‐rump‐length and SIJ size was detected. Similar observations have been reported in other species. In horses, for example, the joint surface area of the SIJ increases from approximately 1.0 cm^2^ in a 10 kg pony foal to around 18 cm^2^ in adult horses weighing 500–600 kg (Dalin & Jeffcott, 1986b). In humans, the joint surface area increases from approximately 1.5 cm^2^ in newborns to about 17.5 cm^2^ in adults (Brooke, 1924). These comparisons suggest that SIJ surface does not necessarily correlate with body size. Additionally, our findings show no sexual dimorphism in joint size in the examined cats. This stands in contrast with findings in humans, where differences in both size and morphology of the auricular surface between females and males have been described (Anastasiou & Chamberlain, 2013; Brooke, 1924; Nishi et al., 2020). One potential explanation for this discrepancy is that sexual dimorphism of the SIJ develops during puberty (Brooke, 1924). As most domestic cats are neutered before or at the onset of puberty, at four to six months of age, the hormonal influences that may contribute to the development of sexual dimorphism could possibly be reduced or absent. This tentatively suggests that the sex‐related differences in SIJ morphology may not be uniformly developed to the same extent across all individuals.

Furthermore, one possible explanation for the absence of significant differences in the mean size of the auricular surface or the total joint surface might be that in the present cohort, animals with relatively larger right or left joint surfaces statistically balance each other, resulting in an overall appearance at the group level. However, examinations at the individual level revealed asymmetries in joint size and the SI of the study population showed a wide range of values. As no comparative threshold exists for the feline SIJ, no clear interpretation or comparison with other studies is not possible in this case. However, low SI values generally indicate a high degree of bilateral symmetry, potentially reflecting balanced anatomical development and uniform load distribution. By contrast, higher SI values signify increasing asymmetry, which may suggest compensatory mechanisms or unilateral joint degeneration from a morphological perspective. It is widely accepted that the biological body is not a perfect mirror image; both organs and skeletal structures naturally exhibit a certain degree of asymmetry. One reason is the body's adaptation to a “dominant” side, that experiences more frequent or intense mechanical loading (Auerbach & Ruff, 2006; Bass et al., 2002; Kontulainen et al., 2002; Nishi et al., 2020; Stirland, 1993; Zhang et al., 2022). Given that the SIJ is the primary transmitter of force from the hindlimbs to the axial skeleton, these discrepancies potentially result from asymmetric loading patterns during high‐impact activities, such as jumping or rapid acceleration. Consequently, an elevated SI does not necessarily indicate a pathological condition but could represent a normal functional adaption to mechanical demands, or be the consequence of a past injury.

In addition, the osseous components of the sacroiliac joint comprise the pelvis and the sacrum. The latter is suspended between the iliac wings and exhibits greater mobility (Kiapour et al., 2020). Due to the sacrum's unique position, it is reasonable that the joint surface of the ilium is slightly larger than that of the sacrum, thus effectively “enclosing” it. The broad attachment of the ventral and dorsal sacroiliac ligaments to the ilium may further explain the relatively larger surface area of the iliac component.

### 4.4 Limitations of the study

When interpreting the results, it is essential to consider the limitations inherent in the study design. The exact age, lifestyle, and medical history of the cats examined were unknown, and the cohort consisted predominantly of neutered animals. This restricts more definitive conclusions that can be drawn regarding functional wear and tear, hormonal influence, or specific functional disorders. Additionally, the manual preparation may have led to the under‐detection of fine nerve branches. The absence of electrophysiological or immunohistochemical confirmation regarding the nerve modality precludes definitive conclusions regarding the exact neuroanatomical subtypes. Furthermore, there may have been slight deviations in the exact position of the cutaneous termination points of the nerve branches, as the tissue was not fixed but thawed, and therefore soft and flexible. Moreover, the classification of morphological forms is by its very nature subjective and could not be statistically controlled due to the lack of assessment of observer error. Finally, the histological evaluation was based on representative samples rather than complete serial sections. Therefore, the possible presence of micro innervation or very small fibers—particularly in the four samples in which no nerve were detected microscopically—cannot be ruled out with certainty.

## 5 CONCLUSIONS

In conclusion, the present study provides two primary contributions to the structural understanding of the SIJ in domestic cats. It indicates that the dorsal sacroiliac ligaments are innervated by the seventh lumbar and first sacral spinal nerves. Additionally, it documents considerable variation in the macroscopic shape of the auricular surface. There were no significant differences observed in the size of the auricular surface or the total joint size between male and female cats.

However, it is imperative to acknowledge that the morphological findings do not provide direct evidence of clinical disease, joint function, or pain. Instead, they are intended to serve as a cautious, descriptive anatomical basis to guide and inform future immunohistochemical, functional, and clinical research regarding the impact of SIJ dysfunction in small animal veterinary medicine.

## AUTHOR CONTRIBUTIONS

Concept/design: AK, NR. Acquisition of data: AK. Data analysis/interpretation: AK, MK, TG, SF, CM, NR. Drafting of the manuscript: AK. Critical revision of the manuscript and approval of the article: AK, MK, TG, SF, CM, NR.

## FUNDING INFORMATION

The research was supported by the Dr. Heidi und Karl‐Heinz Kübler Stiftung.

## CONFLICT OF INTEREST STATEMENT

None of the authors have a conflict of interest to disclose.

## Supporting information


Table S1.

## Data Availability

The data that support the findings of this study are available from the corresponding author upon reasonable request.
