# Supplementary material for: The feline sacroiliac joint: An overview of its innervation and morphology
Source: J Anat. 2026 Oct 2:10.1111/joa.70252. Online ahead of print. doi: 10.1111/joa.70252 (PMC13633417; doi:10.1111/joa.70252)
Supplement: Supplementary file 1 [file JOA-9999-0-s001.docx]

**Suppl. Table 1: Number of cat, breed, weight (kg), crown-rump-length (cm) and gender of adult domestic cats.** N.M.: Neutered male; S.F. Spayed female; F.: Female; Norw. For. Cat: Norwegian forest cat; ESH: European shorthair cat; GLH: German longhair cat

| **Number of cat** | **Breed** | **Weight (kg)** | **Crown-rump-length (cm)** | **Gender** |
| --- | --- | --- | --- | --- |
| 1 | ESH | 4.00 | 41.20 | N.M. |
| 2 | ESH | 3.10 | 45.20 | S.F. |
| 3 | ESH | 2.80 | 42.20 | S.F. |
| 4 | ESH | 3.55 | 43.20 | S.F. |
| 5 | ESH | 3.43 | 42.20 | N.M. |
| 6 | ESH | 3.17 | 36.40 | S.F. |
| 7 | ESH | 2.93 | 41.80 | N.M. |
| 8 | ESH | 3.58 | 41.30 | N.M. |
| 9 | ESH | 2.70 | 39.70 | F. |
| 10 | ESH | 1.84 | 37.00 | N.M. |
| 11 | ESH | 1.95 | 40.80 | N.M. |
| 12 | ESH | 3.81 | 42.40 | N.M. |
| 13 | ESH | 4.20 | 42.30 | N.M. |
| 14 | ESH | 3.28 | 43.20 | S.F. |
| 15 | ESH | 1.70 | 39.40 | S.F. |
| 16 | ESH | 2.35 | 37.20 | S.F. |
| 17 | Norw. For. Cat | 3.35 | 41.50 | S.F. |
| 18 | ESH | 3.55 | 38.30 | S.F. |
| 19 | Birman | 4.95 | 42.20 | N.M. |
| 20 | ESH | 2.65 | 40.20 | N.M. |
| 21 | ESH | 2.20 | 39.90 | N.M. |
| 22 | ESH | 2.35 | 40.70 | N.M. |
| 23 | ESH | 3.95 | 40.20 | N.M. |
| 24 | ESH | 3.75 | 41.20 | S.F. |
| 25 | ESH | 2.80 | 40.00 | N.M. |
| 26 | Persian cat | 3.15 | 39.80 | N.M. |
| 27 | ESH | 3.65 | 42.20 | N.M. |
| 28 | ESH | 2.80 | 42.80 | F. |
| 29 | ESH | 2.12 | 41.70 | S.F. |
| 30 | ESH | 3.29 | 42.90 | N.M. |
| 31 | ESH | 3.62 | 40.20 | S.F. |
| 32 | ESH | 2.90 | 36.30 | F. |
| 33 | Maine-Coon | 4.81 | 43.60 | N.M. |
| 34 | ESH | 3.95 | 39.80 | S.F. |
| 35 | ESH | 2.86 | 37.10 | S.F. |
| 36 | ESH | 3.48 | 40.20 | S.F. |
| 37 | ESH | 5.28 | 45.10 | N.M. |
| 38 | GLH | 3.27 | 35.80 | S.F. |
| 39 | ESH | 4.26 | 40.00 | S.F. |
| 40 | Maine-Coon | 5.47 | 42.20 | N.M. |

**Suppl. Table 2: Two-sample t-test analysis of sex-specific differences in SIJ dimensions between female and male cats.** TJ: Total joint surface; AS: Auricular surface; I: Ilium; S: Sacrum. n=40

| **Parameter** | **Female (mean ± SD)** | **Male (mean ± SD)** | **t-statistic** |
| --- | --- | --- | --- |
| I R AS (mm^2^) | 68.46 ± 10 | 63.46 ± 9 | 0.117 |
| I R TJ (mm^2^) | 186.16 ± 23 | 188.32 ± 22 | 0.766 |
| S R AS (mm^2^) | 64.34 ± 10 | 62.17 ± 8 | 0.446 |
| S R TJ (mm^2^) | 182.52 ± 24 | 184.55 ± 23 | 0.784 |
| I L AS (mm^2^) | 63.15 ± 8 | 64.34 ± 13 | 0.733 |
| I L TJ (mm^2^) | 181.96 ± 27 | 186.208 ± 21 | 0.581 |
| S L AS (mm^2^) | 62. 42 ± 10 | 60.52 ± 14 | 0.631 |
| S L TJ (mm^2^) | 181.23 ± 31 | 184.72 ± 20 | 0.672 |
